# Brain-Targeted Delivery of Trans-Activating Transcriptor-Conjugated Magnetic PLGA/Lipid Nanoparticles

**DOI:** 10.1371/journal.pone.0106652

**Published:** 2014-09-04

**Authors:** Xiangru Wen, Kai Wang, Ziming Zhao, Yifang Zhang, Tingting Sun, Fang Zhang, Jian Wu, Yanyan Fu, Yang Du, Lei Zhang, Ying Sun, YongHai Liu, Kai Ma, Hongzhi Liu, Yuanjian Song

**Affiliations:** 1 Jiangsu Key Laboratory of Brain Disease Bioinformation, Xuzhou Medical College, Xuzhou, Jiangsu Province, China; 2 College of Animal Science and Technology, Yunnan Agricultural University, Yunnan, Kunming Province, China; 3 School of Pharmacy, Xuzhou Medical College, Xuzhou, Jiangsu Province, China; 4 Research Center for Neurobiology and Department of Neurobiology, Xuzhou Medical College, Xuzhou, Jiangsu Province, China; 5 School of Basic Education Sciences, Xuzhou Medical College, Xuzhou, Jiangsu Province, China; 6 Department of Neurology, Affiliated Hospital of Xuzhou Medical College, Xuzhou, Jiangsu Province, China; 7 Department of Medical Information, Xuzhou Medical College, Xuzhou, Jiangsu Province, China; Brandeis University, United States of America

## Abstract

Magnetic poly (D,L-lactide-co-glycolide) (PLGA)/lipid nanoparticles (MPLs) were fabricated from PLGA, L-α-phosphatidylethanolamine (DOPE), 1,2-distearoyl-sn-glycero-3-phosphoethanolamine-N-amino (polyethylene glycol) (DSPE-PEG-NH_2_), and magnetic nanoparticles (NPs), and then conjugated to trans-activating transcriptor (TAT) peptide. The TAT-MPLs were designed to target the brain by magnetic guidance and TAT conjugation. The drugs hesperidin (HES), naringin (NAR), and glutathione (GSH) were encapsulated in MPLs with drug loading capacity (>10%) and drug encapsulation efficiency (>90%). The therapeutic efficacy of the drug-loaded TAT-MPLs in bEnd.3 cells was compared with that of drug-loaded MPLs. The cells accumulated higher levels of TAT-MPLs than MPLs. In addition, the accumulation of QD-loaded fluorescein isothiocyanate (FITC)-labeled TAT-MPLs in bEnd.3 cells was dose and time dependent. Our results show that TAT-conjugated MPLs may function as an effective drug delivery system that crosses the blood brain barrier to the brain.

## Introduction

Developing an effective drug delivery system with the ability of crossing the blood brain barrier (BBB) is the crucial point in treating diseases of the human central nervous system (CNS) effectively. Many potential drugs have been abandoned during their development for their poor ability to cross the BBB in sufficient quantities to produce a therapeutic effect [1]. The BBB is not only an anatomical barrier to the free movement of solutes between the blood and brain, but also a transport and metabolic barrier [2]. Consequently, developing tools and methods that allow the therapeutic agents to delivery to the brain safely and effectively *in* *vivo* is important.

Nanocarrier systems, such as micelles, liposomes [3], [4], and polymeric nanoparticles [5], [6], have been investigated for delivering therapeutic agents to the brain [7]. Magnetically driven Nanocarrier drug delivery systems are powerful tools for delivering drugs, genes and cells to a target organ, and may be suitable for delivering drugs to the brain. These systems have many unique characteristics, including the delivery of a range of biomolecules *in* *vivo*, such as DNA and siRNA [8]; non-invasive magnetic targeting with therapeutic biomolecules by using external magnetic fields over the target organ or injury site, and trackability *in* *vivo* with various imaging systems [9]–[11]. Furthermore, carrying capacity could be improved by magnetic guidance to target the drug to the brain parenchyma. In order to increase the uptake efficiency, magnetic nanoparticles can be modified with specific ligands such as cationic albumin [12], thiamine [13], or transferrin [14], surfactant coatings [15], proteins and peptides [16]–[18]. The HIV-1 trans-activating transcriptor (TAT) peptide [19], which is a cell penetrating peptide, has been widely used to increase the transport efficiency of drug-loaded NPs across the BBB to the CNS [20]. TAT has been used to form the Chitosan-PEG-TAT nanoparticles for complexing siRNA to be delivered in neuronal cells [21]. TAT-GS nanoparticle-mediated calcitonin gene-related peptide (CGRP) gene delivery has been proposed to be an innovative strategy for cerebral vasospasm [22]. Therefore, normal brain drugs combining with peptides, nanotechnology and magnetic targeting may greatly improve the treatment of brain disorders [23].

Magnetic poly (D,L-lactide-co-glycolide) (PLGA)/lipid NPs (MPLs) combine the advantages of PLGA and magnetic liposomes, such as a well-defined biocompatible coating and simple fabrication [24], and have broad applications, including drug delivery and biodetection [25]–[27]. Monodispersed superparamagnetic magnetite/lipid nanospheres have been studied extensively [28]–[30]. Compared with conventional stealth liposomes [31], [32], the surface of MPLs can be easily modified by groups, including functional amino (-NH_2_) headgroups. In this work, we combine magnetic guidance and cell penetrating peptides to improve the delivery of drugs by TAT-conjugated MPLs.

## Materials and Methods

### Materials

Cholesterol and 1,2-distearoyl-sn-glycero-3-phosphoethanolamine-N-[amino (polyethylene glycol)-2000] (DSPE-PEG2000-NH_2_) were purchased from Avanti Polar Lipids (USA). Quantum Dot (QD) were purchased from Wuhan Jiayuan Quantum Dots Co., Ltd (China). TAT-peptide with the sequence CGRKKRRQRRRK was purchased from ShineGene (China). Chitosan (deacetylation >90%, M_w_ = 50,000) was supplied by Yuhuan Aoxing (China). Octadecyl quaternized carboxymethyl chitosan (OQCMC) and hydrophobic magnetic nanoparticles (HMNs) were prepared according to a previously published method [26], [28]. Poly (D,L-lactic-co-glycolic acid) (M_w_ = 10,000, lactic/glycolic acid ratio = 50/50) was purchased from Shandong Key Laboratory of Medical Polymer Material (China). L-α-Phosphatidylethanolamine (DOPE), N-succinimidyl 3-(2-pyridyldithio) propionate (SPDP), 2-iminothiolane, disuccinimidyl suberate (DSS), PD-10 columns were purchased from Sigma-Aldrich (USA). All other chemicals were of reagent grade and were used as received.

### Preparation of MPLs

The MPLs were prepared by the reverse-phase evaporation (REV) method. For blank MPLs, PLGA, OQCMC, HMNs, DOPE, and DSPE-PEG-NH_2_ (weight ratio = 1∶0.2∶0.3∶0.2∶0.2, total weight 30 mg) were dissolved in chloroform (4.0 mL) at room temperature to obtain the organic phase. The aqueous deionized water phase (6.0 mL) was mixed with the organic phase under sonication for 120 s at an output of 100 W. The organic solvents were evaporated on a rotary evaporator to form a gel-like MPL suspension. The MPLs were then separated by using a magnet and were washed with deionized water for three times. The collected product was freeze-dried and stored at 4°C.

For drug-loaded MPLs, the appropriate drug PLGA, OQCMC, HMNs and DSPE-PEG-NH_2_ were dissolved in chloroform. Hesperidin (HES) and naringin (NAR) were soluble in the organic phase whereas glutathione (GSH) was dissolved in aqueous solution. The weight ratio of the drug to PLGA was adjusted according to experimental requirements.

### TAT conjugation via a disulfide linkage

The MPLs were reacted with SPDP to install a 2-pyridyldithiol-end group on the surface of the NPs. A 0.1 M phosphate-buffered saline (PBS; pH 7.4) solution of MPL (5 mg/mL, 2.0 mL) was added to SPDP (2.0 mg) in DMSO (0.32 mL). The mixture was incubated at room temperature for 60 min. Low molecular weight impurities were dialyzed against 0.1 M PBS for 5 h with a dialysis bag (M_w_ = 12,000–14,000).

The 2-pyridyl disulfide-conjugated MPL solution was added to a 0.1 M PBS solution of TAT (5 mg/mL, 1.0 mL). The mixture was incubated overnight at 4°C to form a disulfide linkage between the surface of the MPLs and the TAT peptide. The excess TAT peptide was not removed and the TAT-conjugated MPL solution was stored at 4°C. The P2T method was used to verify the conjugation of the 2-pyridyl disulfide groups to the MPLs. The TAT-conjugated MPL solution was separated with a magnet and the supernatant solution was collected. The supernatant (300 µL) was diluted to 3 mL with PBS. UV-Visible Spectrophotometer was used to measure the absorbance of the MPL samples at 343 nm.

### Characterization of MPLs

The particle size of the MPLs in solution was measured by quasielastic laser light scattering with a zeta potential analyzer. The MPL concentration in PBS was 0.3 mg/mL. Each measurement was repeated three times, and an average value was used. The size measurements were performed by multimodal analysis.

The morphologies of MPLs were observed by atomic force microscopy. For the AFM observations, droplets of the samples (approximately 30 µL) were deposited onto freshly cleaved mica and left for 10 min. Images were captured with scan rates between 0.5 and 1 Hz. The magnetic properties of magnetic TAT-MPLs were determined with a vibrating sample magnetometer.

### Drug loading efficiency and release *in* *vitro*

The concentration of the three drugs was determined by UV/Vis spectroscopy at a fixed wavelength. The calibration curve was obtained in the concentration range of 0.5–50 µg/mL and the detection limit was 0.05 µg/mL.

After preparing the drug-loaded MPLs, the solution was centrifuged to remove any unencapsulated drug (4,000 rpm for 10 min). Drug content was determined by calculating the weight of the drug encapsulated in the MPLs.

The drug encapsulation efficiency (EE) and drug loading efficiency (LE) of the process were calculated using the following equation:
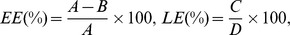
where A is the total amount of drug; B is the amount of unencapsulated drug; C is the weight of drug in vesicle; D is the weight of vesicle.

*In vitro* drug release experiments of drug-loaded MPLs were carried out under shaking at 100 rpm and 37±0.5°C. The drug-loaded samples were enclosed in a dialysis membrane and then incubated in Phosphate buffer saline (10 mL, pH = 7.4). The buffer solution was exchanged completely at regular time points. The amount of drug release was determined by UV-Visible Spectrophotometer at set times, with PBS as a reference.

### Cell culture and cytotoxicity studies

Bend.3 cells were grown in Dulbecco Modified Eagle Medium supplemented with 10% (v/v) fetal bovine serum and 1% (v/v) penicillin/streptomycin. The cytotoxicity was evaluated by MTT assays. bEnd.3 cells were seeded at a density of 1×10^4^ cells/well in 96-well flat-bottomed microassay plates and incubated for 24 h at 37°C, in a fully humidified atmosphere of 5% CO_2_. An increasing amount of drug-loaded MPLs and TAT-MPLs (1–100 µg/mL) were added and incubated for 12 h, 24 h and 48 h at 37°C. MTT saline solution (5 mg/mL, 100 µL/well) was added to the cells and formazan crystals were allowed to form over 3 h, then the crystals were dissolved with DMSO. The absorbance was measured at 490 nm with a Multi-Mode Microplate Reader.

### Uptake studies

The uptake and intracellular distribution of QD-loaded fluorescein isothiocyanate (FITC)-TAT-MPLs in bEnd.3 cells were determined qualitatively using confocal fluorescence microscopy (Leica TCS SP8 MP, Leica Micosystems, Germany). bEnd.3 cells were seeded at a density of 8×10^4^ cells/well in a 24-well plate with a pre-sterilized cover glass at the bottom and the cells were allowed to attach overnight. The following day, the cells were treated with QD-loaded FITC-MPLs and QD-loaded FITC-TAT-MPLs in the medium at a concentration of 20 µg/mL. At 0.5, 3, and 12 h after treatment, the cells were washed three times with PBS and fixed with 4% (w/w) paraformaldehyde in PBS for 15 min. The cells were counterstained with 4′,6-diamidino-2-phenylindole (DAPI) and rinsed three times. The images were captured with a confocal laser scanning microscope with appropriate filters for the red QD fluorescence (excitation at 560–575 nm and emission at 605 nm), green FITC fluorescence (excitation at 480–495 nm and emission at 525 nm) and blue DAPI fluorescence (excitation at 358 nm and emission at 461 nm). Finally, images captured using red and blue filters were overlain to determine the localization and association of red QD fluorescence in the cytoplast or nucleus, respectively.

### Qualitative images and quantification of drug-loaded MPLs internalization

To measure the internalization of QD-loaded TAT-MPLs quantitatively, bEnd.3 cells were cultured on 24-well plates for 24 h to achieve approximately 80% confluence. Free FITC, free QDs, QD-loaded FITC-MPLs, or QD-loaded FITC-TAT-MPLs were then added to the wells. After incubation for 0.5, 3, or 12 h the cells were collected for fluorescence measurements (FITC and QDs). The fluorescence from individual cells was detected with a flow cytometer (FACS Calibur, BD Biosciences, USA). To detect the green FITC fluorescence, excitation was with the 488 nm line of an argon laser, and the emission fluorescence was measured at 525 nm. To detect red QD fluorescence, excitation was with the 570 nm line of an argon laser, and the emission fluorescence was measured at 605 nm.

For quantitative analysis, the uptake of FITC and QDs in bEnd.3 cells was determined by using fluorescence and the bicinchoninic (BCA) method. At the end of the treatment period, the cells were washed three times with PBS (pH 7.4) and then incubated with cell culture lysis reagent (50 µL) for 10 min at 37°C. The protein content of the cell lysate was determined using the Pierce BCA protein assay. Cell lysates were harvested by adding PBS (50 µL, pH 7.4) and shaking at 37°C for 2 h. Samples were centrifuged at 10,000 rpm for 10 min at 4°C. The concentrations of the fluorescent materials (FITC and QDs) in the PBS extract were determined by a microplate spectrophotometer. Data are expressed as the amount of fluorescence material normalized to the total cell protein.

### Statistical analysis

All data are expressed as mean±standard error of means. Statistical analyses were performed using Student’s T-test. The differences were considered significant for p values<0.05.

## Results and Discussion

### Formulation of magnetic PLGA/lipids NPs

**Figure 1:**
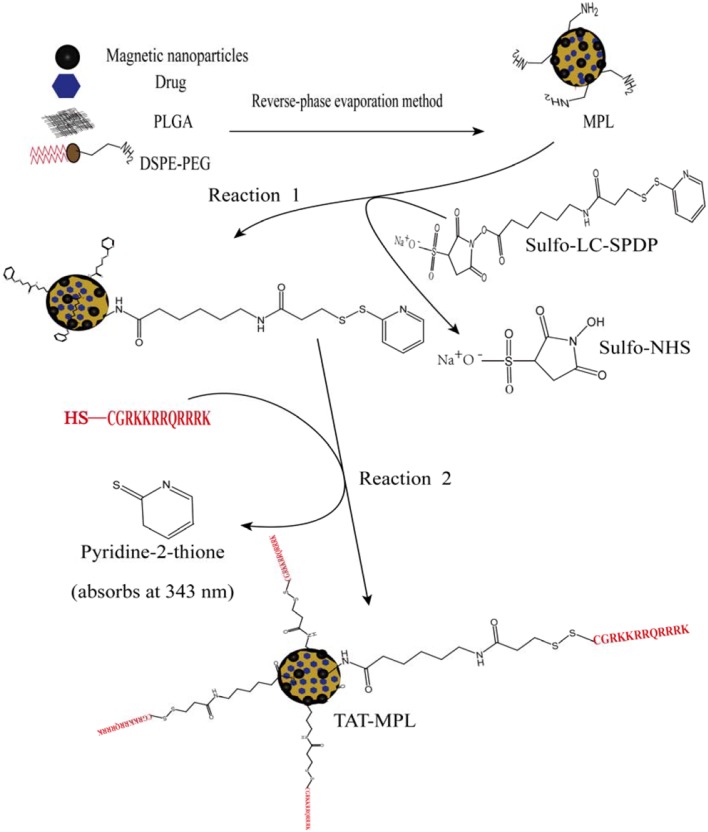
Schematic of the preparation of stealth MPLs and the conjugation of TAT peptide to MPLs.

PLGA/lipid complexes have many advantages as delivery vehicles. They combine the properties of liposomes and PLGA and can be modified with molecular targeting factors. Multifunctional targeted drug delivery systems, such as magnetic cationic liposomes [30] and PEG- and TAT-conjugated MPLs, can be fabricated from DSPE-PEG or DOPE. Attaching flexible PEG polymers to the liposome surface increases the blood circulation time significantly. These PEG-coated liposomes are referred to as stealth liposomes [31], [32]. A schematic illustration of the preparation of stealth TAT-conjugated MPLs is presented in Figure 1. PEGylated MPLs can be assembled by the REV method from DSPE-PEG-NH_2_, OQCMC, and DOPE. The DSPE-PEG-NH_2_ and OQCMC components of MPLs mean that amine groups are present on the surface of stealth MPLs [33]–[35]. In this study, SPDP reagents, which are a unique group of amine- and sulfhydryl-reactive heterobifunctional cross-linkers, were used to form amine-to-sulfhydryl cross-links between molecules. Because TAT peptide contains sulfhydryl groups (-SH), the MPLs are modified by the SPDP reagent in reaction 1. Reaction 2 results in the displacement of a pyridine-2-thione group, the concentration of which may be determined by measuring the absorbance at 343 nm.

**Figure 2:**
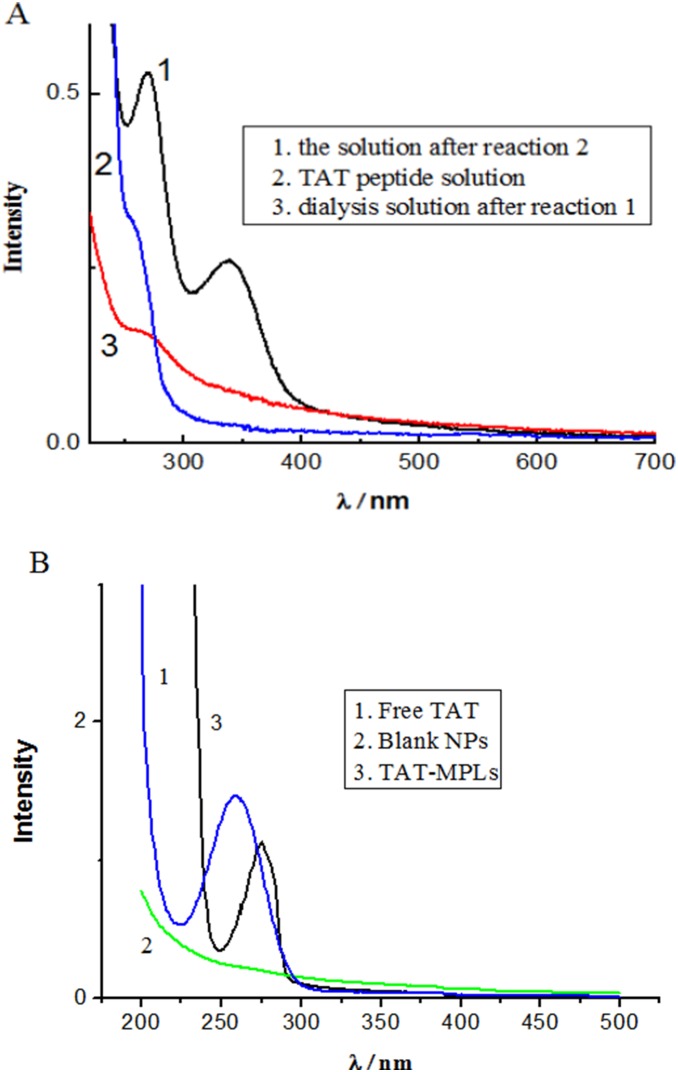
UV/Vis spectra. (A) UV/Vis spectra of the reaction solutions (conjugation of TAT to MPLs). (B) UV/Vis spectra of blank NPs and TAT-MPLs.

UV/Vis spectra of the reaction solutions are shown in Figure 2A. An absorbance peak at 343 nm was clearly visible after reaction 2, which confirmed the presence of pyridine-2-thione. In addition, the peak at 343 nm was not observed for the dialyzed solution after reaction 1 and the TAT peptide solution, indicating that pyridine-2-thione must be the product of reaction 2. The results also suggest that amine groups were present on the surface of MPLs. UV/Vis spectroscopy was also used to determine the content of TAT peptide on the MPLs (Figure 2B). Differences were observed in the UV absorption of MPLs and TAT-MPLs. TAT had an absorption peak at 275 nm. The UV spectrum of the TAT-MPLs was similar to that of TAT, although it also contained a bathochromic shift at 277.5 nm, which was attributed to the addition of TAT to the MPLs. These results indicate that the modification of the TAT unit was responsible for the differences between the UV spectra of MPLs and TAT-MPLs.

### Characterization of MPLs

**Figure 3:**
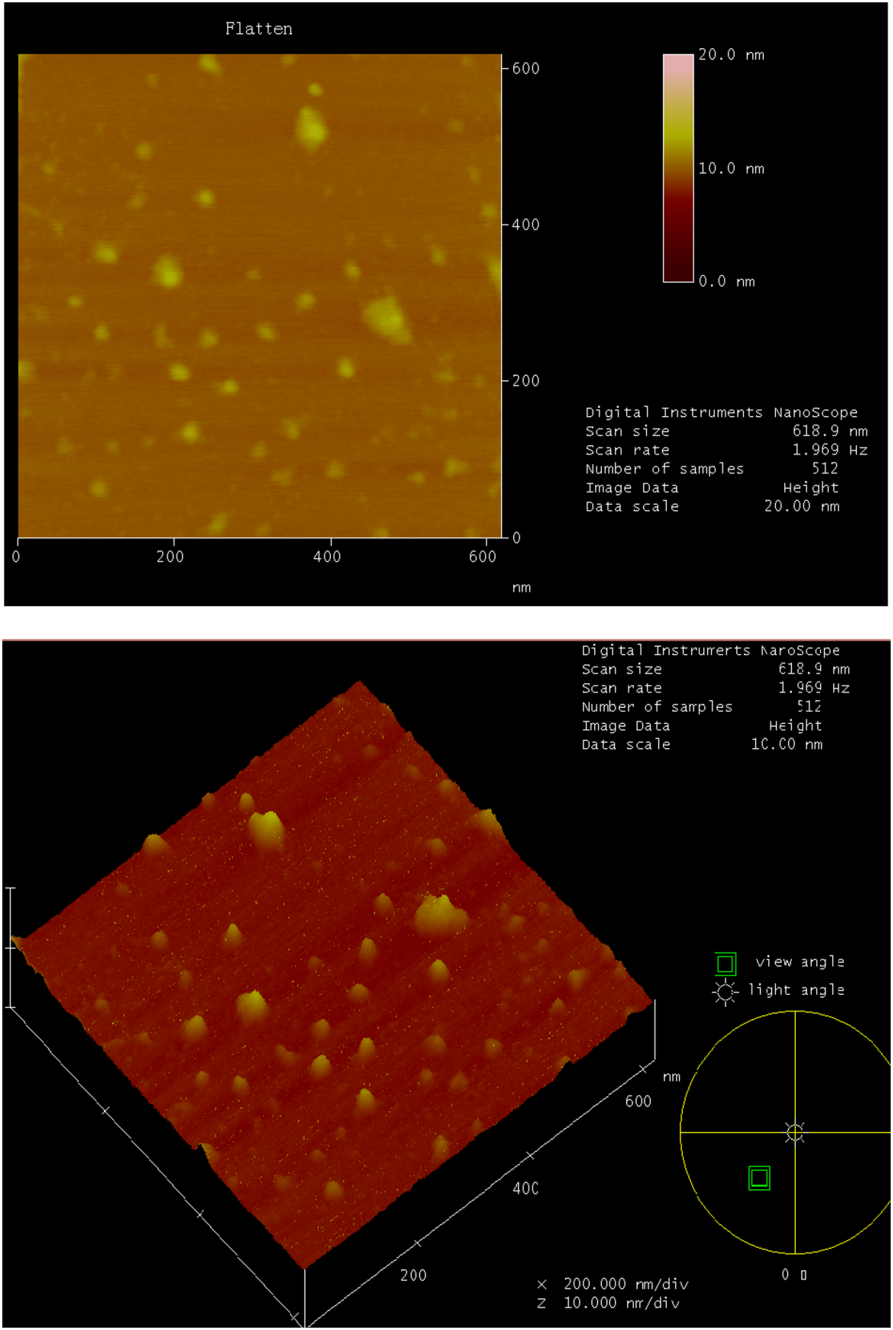
AFM micrographs of TAT-MPLs.

**Figure 4:**
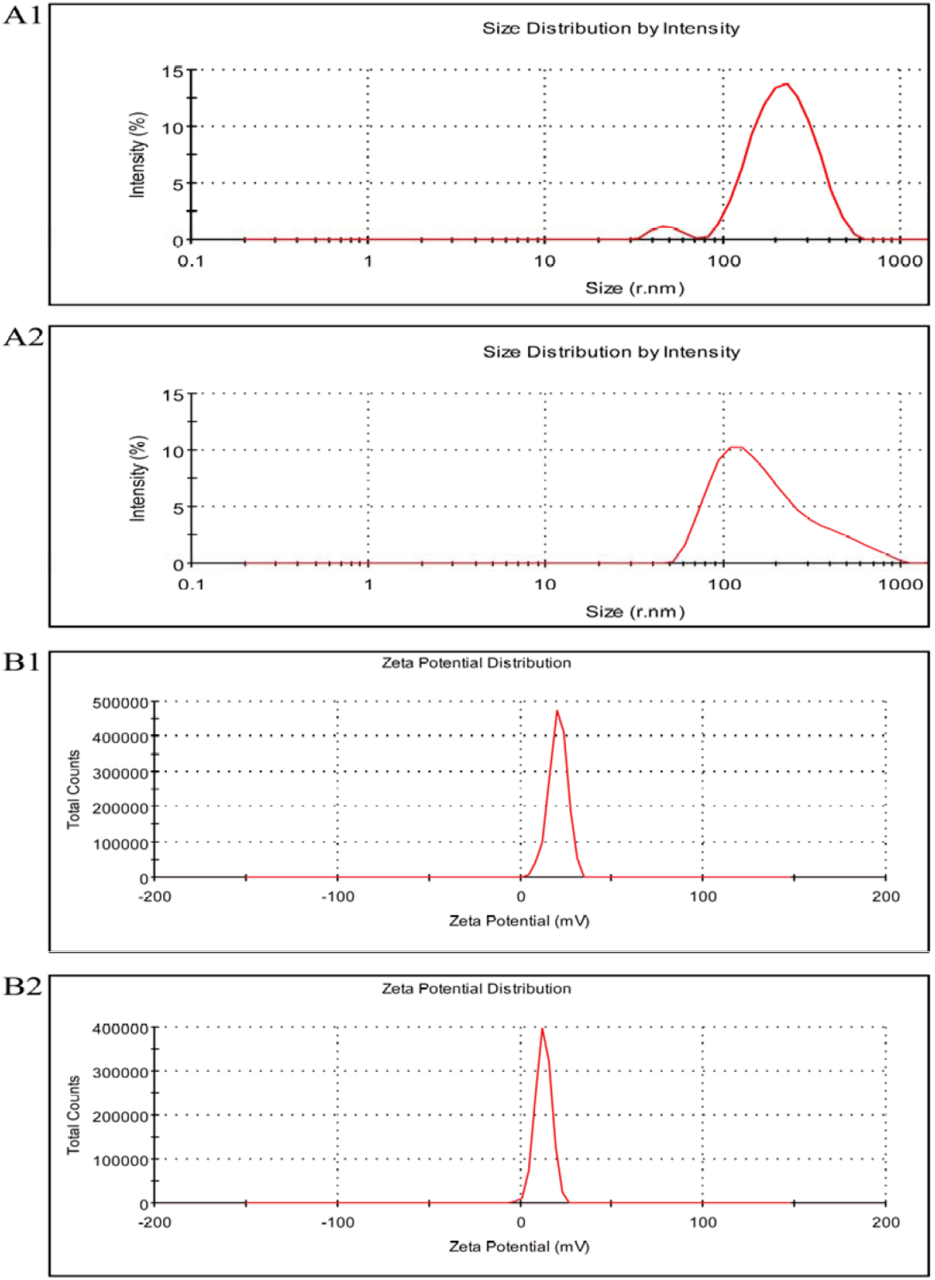
XRD spectrum and Magnetization curve. (A) XRD spectrum of TAT-MPLs. (B) Magnetization curve of Fe_3_O_4_ ferrofluid (1) and TAT-MPLs (2).

The TAT-MPLs generally had irregular spherical shapes (Figure 3) and were about 80 nm in size. The hydrodynamic diameter of TAT-conjugated MPLs was 102.0±0.7 nm, with a corresponding polydispersity index (PDI) of 0.304, which was greater than the AFM diameter because of the hydration of MPLs in aqueous solution. The TAT conjugation decreased the zeta potential of NPs (Figure 4 and Table 1). HES were encapsulated effectively in the TAT-conjugated MPLs, and the drug encapsulation efficiency and loading capacity were above 90% and 10%, respectively. The size of the HES-loaded TAT-conjugated MPLs in PBS solution was 112.9±1.1 nm with a narrow size distribution and a polydispersity index of 0.220 (Table 1). The AFM images also show the spherical shape and homogeneous size distribution of the magnetic NPs.

**Table 1:** Physical characterization of TAT-conjugated MPLs.

Formulation	Mean diameter (nm)	Polydispersity (µ/Γ^2^)	Zeta potential (mV)
MPLs	102.6±1.3	0.202	20.7±3.62
TAT-MPLs	102.0±0.7	0.304	12.7±1.82
GSH-loaded TAT-MPLs	131.8±11.2	0.556	31.7±4.53
HES-loaded TAT-MPLs	112.9±1.1	0.220	5.39±0.42
NAR-loaded TAT-MPLs	93.1±1.6	0.237	32.8±2.43

**Figure 5:**
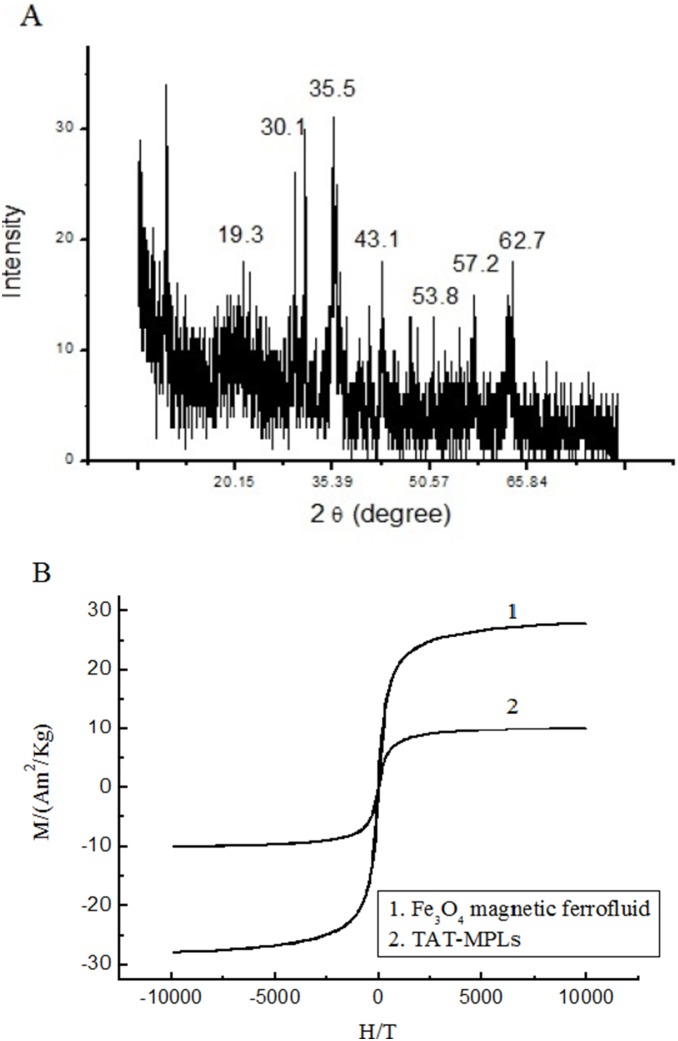
Particle-size-distribution and Zeta potential. (A) Particle-size-distribution based intensity of MPLs (1) and TAT-conjugated MPLs (2) in PBS. (B) Zeta potential of MPLs (1) and TAT-conjugated MPLs (2) in PBS.

X-ray powder diffraction analysis (XRD) is a kind of useful method to demonstrate the structure of magnetic nanoparticles. The XRD pattern of TAT-MPLs was detected as illustrated in Figure 5a. The characteristic peaks of Fe_3_O_4_ ferrofluid are at 2*θ* = 30.1°, 35.5°, 43.1°, 53.7° and 62.7°, which corresponding to crystal face of (220), (311), (400), (422), (511) and (440). The results indicated that TAT-MPLs has the same crystalline structure with Fe_3_O_4_ ferrofluid. The broad peak in the 2*θ* = 15°–25° region approve the existence of PLGA polymer. Figure 5b shows the room-temperature magnetization curves of Fe_3_O_4_ ferrofluid (1) and TAT-MPLs. As shown in the figure, both the samples show a typical superparamagnetic behavior at room temperature without any hysteresis loop. The saturation magnetization value of TAT-MPLs is 10.1 emu/g at 300 K, which is about 36.2% of the magnetization of Fe_3_O_4_ ferrofluid.

**Figure 6:**
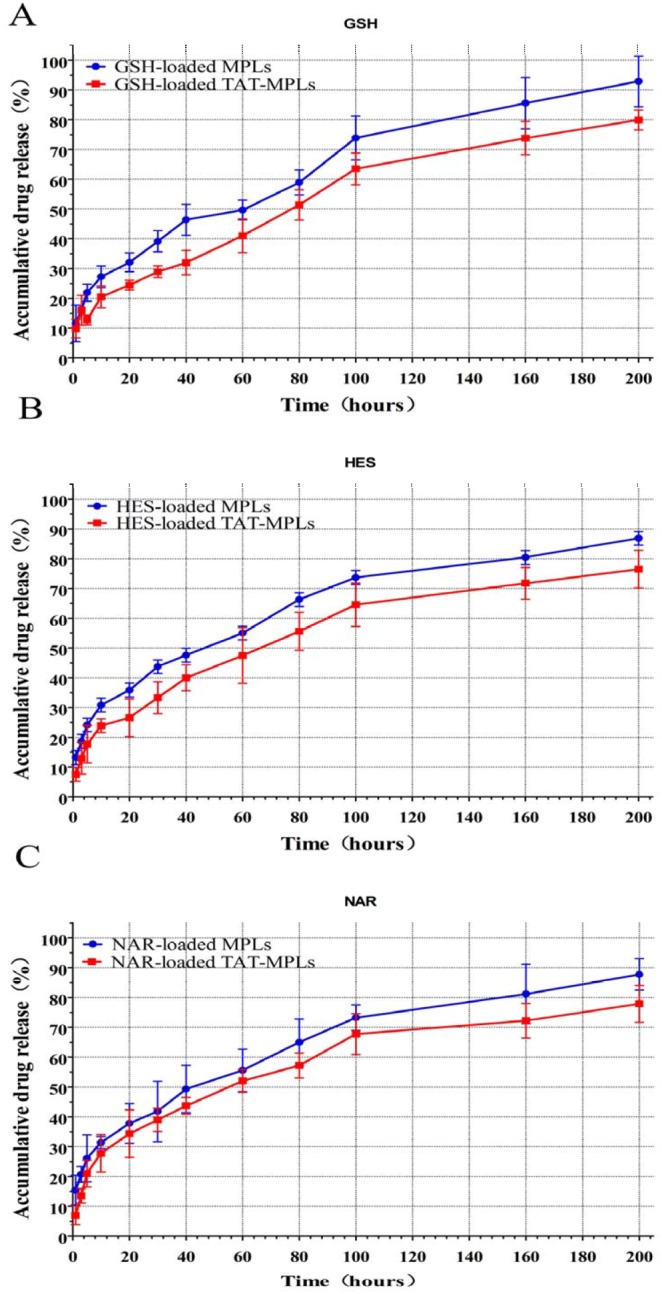
Drug release profiles of the TAT-conjugated MPLs in PBS (pH 7.4) at 37±0.5°C *in* ***vitro.***

The drug release profiles of TAT-conjugated MPLs showed a high burst release during the first 24 h followed by slower release (Figure 6). During the initial burst release, 20%–40% of the encapsulated drug was released from the TAT-conjugated MPLs. After 8 days, 70%–90% of the total drug was released. The surface conjugation of the TAT-MPLs meant that they had a slower drug release than the MPLs.

### Cytotoxicity and growth inhibition of bEnd.3 cells *in* *vitro*

**Figure 7:**
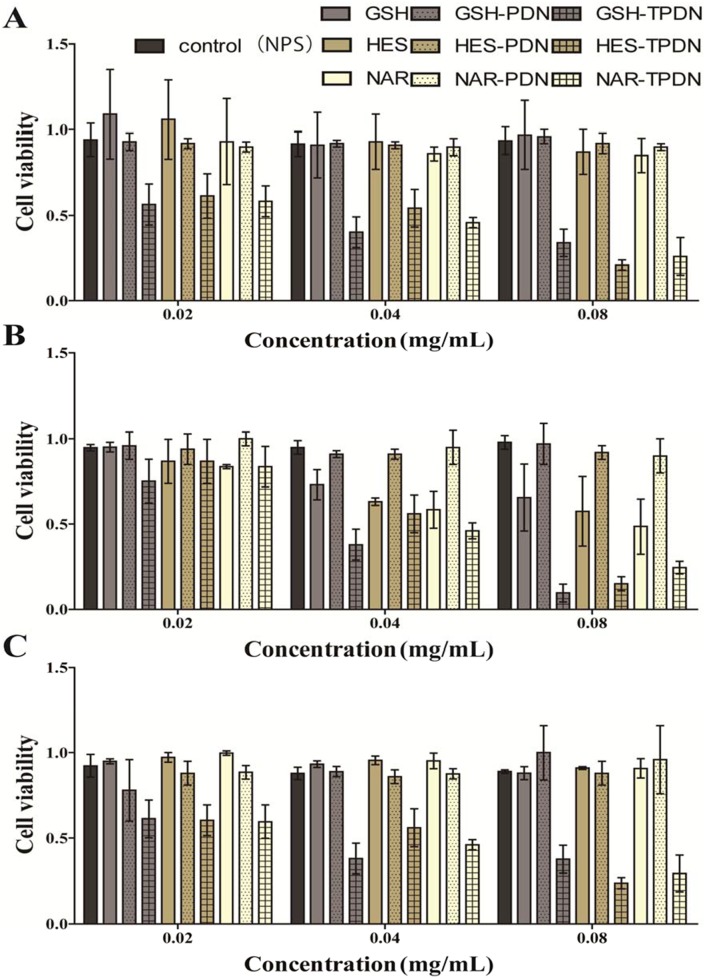
Cytotoxicity of MPLs and drug-loaded TAT-MPLs measured using MTT assays after. (A) 12, (B) 24, and (C) 48 h incubation with bEnd.3 cells. (GSH: glutathione, HES: hesperidin, NAR: naringin).

The cytotoxicity of MPLs and TAT-MPLs in bEnd.3 cells was examined with an MTT assay. Figure 7 shows the cytotoxicity of MPLs and TAT-MPLs at concentrations of less than 100 µg/mL. The bEnd.3 cells treated with MPLs at concentrations of 20.0, 40.0, and 80.0 µg/mL showed good viability (>85%), whereas the cells treated with TAT-MPLs showed poor viability (<75%) after incubation for 12, 24, and 48 h. As the concentration of NPs increased, the drug-loaded TAT-MPLs were more cytoxic than the MPLs. This was because the TAT conjugation delivered a greater amount of the drugs into cells. Furthermore, the cytotoxicity of the blank TAT-MPLs was low and this was confirmed by observing morphological changes in the cells by microscopy. These results demonstrate that TAT-MPLs has low cellular toxicity and were suitable for use in experimental concentrations.

Figure 7 also shows the growth inhibition of bEnd.3 cells by GSH, HES, and NAR in MPLs, TAT-MPLs, and in solution after 48 h at concentrations of GSH, HES, and NAR from 20 to 80 µg/mL. The cell growth inhibition rate was dose-dependent in all the experiments. At GSH, HES, and NAR concentrations of 20 µg/mL, there was no difference in the growth inhibition of bEnd.3 cells treated with the drug solutions and drug-loaded NPs. As the concentration of the drugs increased, the cytotoxicity of the TAT-MPLs was significantly higher (*P*<0.05 for NPs concentrations of 40 and 80 µg/mL). The cell growth inhibition of the TAT-MPLs was also greater than that of the MPLs without TAT, indicating that a greater number of drug-loaded TAT-MPLs entered the cells because TAT increased the cell membrane penetration.

### Intracellular distribution of QD-loaded NPs

**Figure 8:**
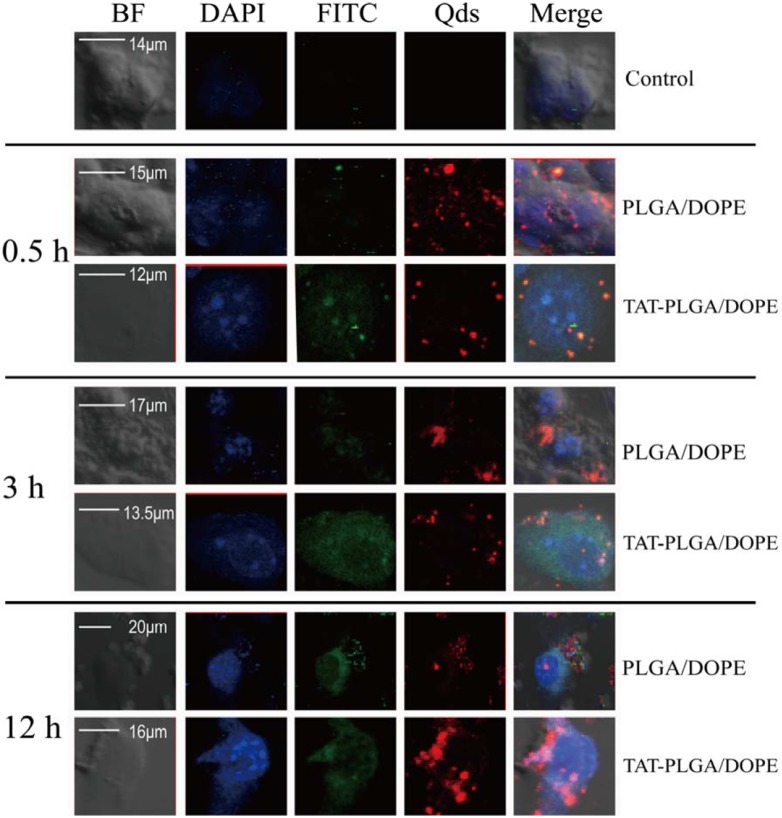
Localization and distribution of QDs encapsulated in TAT-MPLs in bEnd.3 cells. Cells were cultured in FITC-labeled NP-containing medium (1 µM QDs) on a glass-bottomed culture plate for 0.5, 3, and 12 h, treated with DAPI for 5–10 min, and then examined by confocal microscopy.

To verify whether encapsulating different drugs in the NPs affected their trafficking in tumor cells, we evaluated the intracellular distribution of free FITC/QDs, QD-loaded FITC-MPLs, and QD-loaded FITC-TAT-MPLs in bEnd.3 cells with a nuclear stain. The intracellular trafficking of FITC and QDs was studied by laser confocal microscopy to clarify the mechanism of efficacy of drug-loaded NPs. The QD-loaded FITC-NPs showed a diffuse distribution within the cells, with a significant fraction appearing in the cytoplasm near the cell nucleus at 0.5 h. A significant proportion of QD-loaded FITC-NPs also appeared in the cytoplasm. Therefore, the strong fluorescence of TAT-MPLs in the cytoplasm and cell nucleus demonstrates that they can deliver QDs efficiently to bEnd.3 cells. In addition, the FITC-TAT-MPLs were concentrated in the perinuclear region rather than at the cell periphery at 12 h. QDs were also present in the nuclei of cells treated with FITC-TAT-MPLs at 12 h. (Figure 8).

Interestingly, the cells treated with FITC-TAT-MPLs accumulated QDs in the nucleus, whereas cells treated with FITC-MPLs did not. The nuclear delivery of QDs by TAT-MPLs was greater than that by MPLs, which is also consistent with the greater cytotoxicity observed in the MTT assay. These results were further verified by quantitative analysis of accumulation of FITC and QDs.

### Quantitative analysis of accumulation in bEnd.3 cells

**Figure 9:**
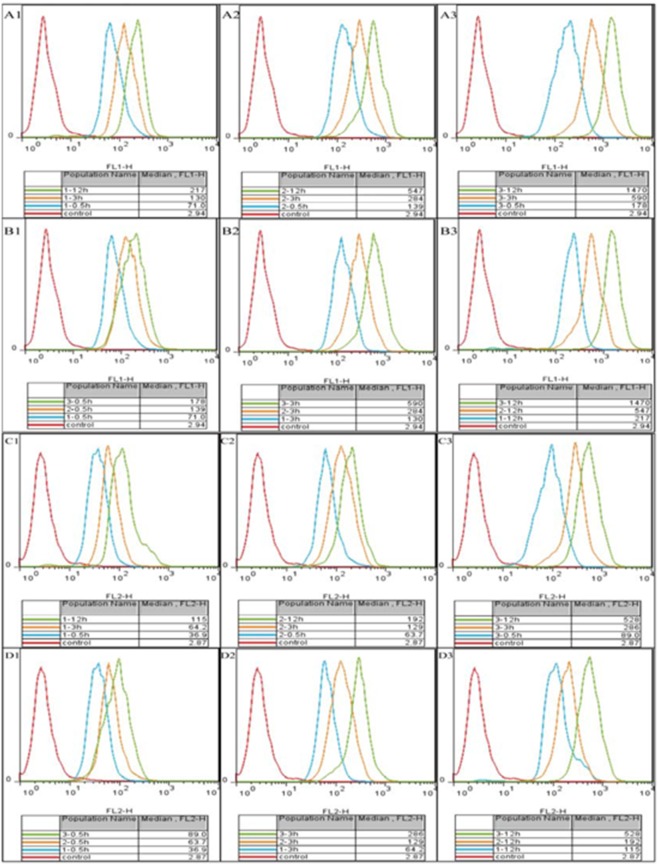
Flow cytometry analysis of bEnd.3 cells. Cells incubated with free FITC and QDs (1), MPLs (2), and TAT-MPLs (3) for 0.5 h, 3 h and 12 h at a NP concentration of 20 µg/mL. The NPs were labeled with FITC. The FITC (A and B) and QD fluorescence intensity (C and D) are shown.

**Figure 10:**
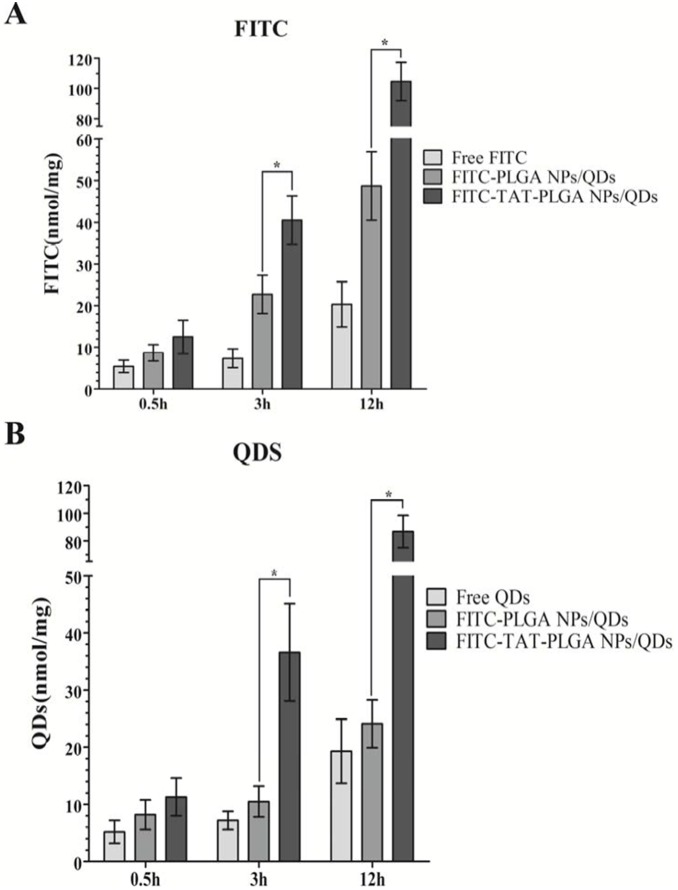
Quantitative analysis of QD-loaded FITC-MPLs and FITC-TAT-MPLs in bEnd.3 cells. Cells were cultured in a 24-well plate for 0.5 h, 3 h and 12 h, lysed, and the FITC and QDs fluorescence were measured by a microplate spectrophotometer to determine FITC (A) and QDs (B) contents in the cells.

To determine the efficacy of cellular drug delivery by TAT-MPLs, the cellular accumulation of equivalent doses of FITC and QDs was quantitatively analyzed in bEnd.3 cells. Figure 9 and Figure 10 show that cells treated with QD-loaded TAT-MPLs accumulated higher levels of QDs than those treated with QD-loaded MPLs. Furthermore, bEnd.3 cells treated with TAT-MPLs accumulated a significantly higher level of QDs and FITC than those treated with QDs and FITC in MPLs and in solution (*P*<0.05) at 3 h and 12 h. Thus, the accumulation of QDs and FITC in bEnd.3 cells is enhanced by TAT conjugation.

Our results show that NPs enhance QDs accumulation considerably in bEnd.3 cells compared with free QDs, and that TAT-MPLs increase QDs accumulation more efficiently than MPLs. These results were confirmed by laser confocal microscopy. Many studies have shown that the increased level of drug accumulation in cells treated with MPLs contributes to the enhanced therapeutic efficacy of drug-loaded NPs [15]. Thus, our method of fabricating TAT-conjugated magnetic NPs successfully produced TAT-MPLs that may be effective for delivering drugs across the BBB.

## Conclusion

Previous studies indicate that there are many applications of magnetic nanoparticles for cells and bacteria [36]–[38]. In this study, we fabricated TAT-conjugated MPLs from OQCMC, DOPE, and magnetic NPs. The size of the HES-loaded TAT-MPLs in aqueous solution was close to 112.9±1.1 nm with a narrow size distribution with a polydispersity index of 0.220. AFM images showed the TAT-MPLs were spherical and had a homogeneous size distribution. The TAT-MPLs showed very strong fluorescence in the cytoplasm and cell nucleus, and delivered QDs to bEnd.3 cells efficiently. The levels of QDs and FITC that accumulated in bEnd.3 cells were dose- and time-dependent for MPLs and TAT-MPLs. The TAT conjugation of MPLs could significantly enhance the cellular delivery and the therapeutic efficacy of drugs in bEnd.3 cells by penetrating the cell membrane. This may be useful in designing drug-loaded NPs for crossing the BBB and delivering drugs to the brain.
